# Performance of DeepSeek and GPT Models on Pediatric Board Preparation Questions: Comparative Evaluation

**DOI:** 10.2196/76056

**Published:** 2025-08-27

**Authors:** Masab Mansoor, Andrew Ibrahim, Ali Hamide

**Affiliations:** 1 Louisiana Campus, Edward Via College of Osteopathic Medicine, 4408 Bon Aire Dr, Monroe, LA, 71203, United States, 1 5045213500; 2 School of Medicine, Texas Tech University Health Sciences Center, Lubbock, TX, United States

**Keywords:** artificial intelligence, large language models, medical education, pediatrics, board examination, DeepSeek, ChatGPT

## Abstract

**Background:**

Limited research exists evaluating artificial intelligence (AI) performance on standardized pediatric assessments. This study evaluated 3 leading AI models on pediatric board preparation questions.

**Objective:**

The aim of this study is to evaluate and compare the performance of 3 leading large language models (LLMs) on pediatric board examination preparation questions and contextualize their performance against human physician benchmarks.

**Methods:**

We analyzed DeepSeek-R1, ChatGPT-4, and ChatGPT-4.5 using 266 multiple-choice questions from the 2023 PREP Self-Assessment. Performance was compared to published American Board of Pediatrics first-time pass rates.

**Results:**

DeepSeek-R1 exhibited the highest accuracy at 98.1% (261/266 correct responses). ChatGPT-4.5 achieved 96.6% accuracy (257/266), performing at the upper threshold of human performance. ChatGPT-4 demonstrated 82.7% accuracy (220/266), comparable to the lower range of human pass rates. Error pattern analysis revealed that AI models most commonly struggled with questions requiring integration of complex clinical presentations with rare disease knowledge.

**Conclusions:**

DeepSeek-R1 demonstrated exceptional performance exceeding typical American Board of Pediatrics pass rates, suggesting potential applications in medical education and clinical support, though further research on complex clinical reasoning is needed.

## Introduction

The integration of artificial intelligence (AI) in medical education and assessment raises important questions about the capabilities of large language models (LLMs) in understanding and applying pediatric knowledge. Recent advancements in AI have produced models with increasingly advanced medical reasoning capabilities [1,2], but limited research exists evaluating AI performance on standardized medical assessments. This study evaluates the performance of 3 leading LLMs (DeepSeek-R1 [DeepSeek AI, 2024], ChatGPT-4 [OpenAI, 2023], and ChatGPT-4.5 [OpenAI, 2024]) on a set of 2023 pediatric board examination preparation questions (2023 PREP Self-Assessment, American Academy of Pediatrics), a comprehensive resource containing case-based multiple-choice questions designed to simulate actual board examinations [3]. We hypothesized that newer AI models would demonstrate improved accuracy on pediatric knowledge assessment, potentially approaching the performance levels of board-certified pediatricians taking certification examinations.

## Methods

### Overview

We conducted a comparative analysis of 3 advanced LLMS (DeepSeek-R1, ChatGPT-4, and ChatGPT-4.5) using a set of 266 questions from the 2023 PREP Self-Assessment from the American Academy of Pediatrics. In compliance with fair use copyright law and with methods deemed exempt by the Healthy Steps Pediatrics Ethics Committee, we entered 266 questions and answer choices from the Pediatrics 2023 PREP Self-Assessment into the 3 LLM platforms. DeepSeek-R1 (DeepSeek AI, 2024), ChatGPT-4 (OpenAI, 2023, gpt-4-turbo, 128k context window), and ChatGPT-4.5 (OpenAI, 2024, gpt-4.5-turbo, 128k context window) were accessed through their respective web interfaces in February 2025.

The 2023 PREP Self-Assessment was selected as it represents the most comprehensive and current pediatric board preparation resource, designed by the American Academy of Pediatrics to mirror the content, format, and difficulty of actual American Board of Pediatrics (ABP) examinations. The questions cover all major pediatric domains in proportions similar to the ABP content outline. The use of PREP questions was determined to constitute fair use for research purposes under 17U.S.C. §107, considering (1) noncommercial educational purpose, (2) factual nature of test questions, (3) limited amount used (266 of thousands of available questions), and (4) no market harm to the copyright holder. Questions were entered manually without reproducing answer explanations or proprietary content. As a subscription-based resource, the likelihood of PREP questions appearing verbatim in training datasets is low. However, we acknowledge that similar pediatric medical knowledge exists in publicly available resources like medical textbooks and journals that may have been included in model training.

Each AI model was presented with identical questions in their original multiple-choice format. All questions were text-based without images or clinical photographs. Each model was queried using standardized prompts: “Please answer the following multiple-choice question by selecting the best answer: [question text].” Default temperature settings were used (temperature=1.0 for ChatGPT models, default settings for DeepSeek-R1). No chain-of-thought or multistep reasoning prompts were used to maintain consistency across models. All queries were performed once without retries. Questions were presented sequentially without access to previous answers. Responses were collected and evaluated against the established correct answers. Performance was measured by calculating the percentage of correct responses for each model. In addition, 95% confidence intervals were calculated using the Wilson score method. Model performance differences were assessed using the McNemar test for paired comparisons.

To contextualize these findings, we compared the AI models’ performance to published data on first-time pass rates for board-certified pediatricians taking the ABP examination. This comparison provides a benchmark for evaluating the clinical relevance of AI performance in pediatric knowledge assessment. It is important to note that the human percentages reported by the ABP represent pass rates—the proportion of examinees who achieve or exceed the passing threshold in a given year—rather than the raw percentage of questions answered correctly. The ABP does not publicly release its exact passing cutoff, but historical reports and candidate feedback suggest that it is approximately equivalent to answering about 70% of questions correctly [4]. Successful test takers often score well above this minimum, with average performance typically exceeding 80%. Therefore, while AI model performance in this study is expressed as the percentage of correct responses, the human figures used for comparison reflect an outcome-based measure (pass/fail) rather than direct accuracy.

### Ethical Considerations

The Healthy Steps Pediatrics Ethics Committee is an institutional committee that evaluates research proposals within our affiliated private practice network. This committee consists of 3 board-certified pediatricians who review research for ethical considerations. The committee determined this study was exempt from formal institutional review board approval as it involved publicly available AI tools and did not include human subjects or protected health information.

## Results

The 3 AI models demonstrated marked differences in performance when tested on 266 pediatric board examination preparation questions. DeepSeek exhibited the highest accuracy at 98.1% (95% CI 95.7%‐99.4%; 261/266 correct responses), outperforming both ChatGPT models (Table 1). ChatGPT-4 achieved an accuracy of 82.7% (95% CI 77.7%‐87.0%; 220/266 correct responses), while ChatGPT-4.5 showed improvement over its predecessor, with approximately 96.6% accuracy (95% CI 93.7%‐98.4%), missing only 9 questions. The difference between DeepSeek-R1 and ChatGPT-4.5 was not statistically significant (*P*=.38, McNemar test).

Error pattern analysis revealed that AI models most commonly struggled with questions requiring integration of complex clinical presentations with rare disease knowledge (Table 2). For example, DeepSeek’s 5 incorrect answers primarily involved metabolic disorders and rare genetic syndromes, particularly questions requiring correlation between subtle biochemical abnormalities and uncommon clinical presentations. ChatGPT models additionally struggled with complex medication dosing calculations and interpretation of pediatric growth parameters in the context of genetic disorders. Notably, there was minimal overlap in the specific questions missed by each model, suggesting that different LLMs have distinct knowledge gaps despite similar training paradigms.

**Table 1.** Performance of large language models on 2023 Pediatric Board Examination Preparation Questions.^a^

Artificial intelligence model	Correct answers	Accuracy (%)	Comparison to ABP^b^ pass rates^c^
Deepseek-R1	261	98.1	Exceeds typical ABP pass rate
ChatGPT-4.5	257	96.6	Upper threshold of ABP pass rate
ChatGPT-4	220	82.7	Comparable to lower range of ABP pass rate

a Each model was tested on 266 multiple-choice questions from the American Academy of Pediatrics 2023 PREP Self-Assessment. Accuracy was calculated as the percentage of correct responses. Performance is contextualized relative to the typical first-time pass rates (80%‐89%) for board-certified pediatricians on the ABP examination. DeepSeek-R1, ChatGPT-4, and ChatGPT-4.5 were tested on identical questions. Pass rates represent historical ABP first-time exam performance.

b ABP: American Board of Pediatrics.

c ABP first-time pass rates for board-certified pediatricians typically range from 80%‐89% (80% in 2022 and 89% in 2024 for general pediatrics) [5].

**Table 2.** Error pattern analysis by knowledge domain.^a^

Knowledge domain	DeepSeek-R1 (N=5), n (%)	ChatGPT-4.5 (N=9), n (%)	ChatGPT-4 (N=46), n (%)
Metabolic disorders	3 (60)	4 (44)	15 (33)
Rare genetic syndromes	2 (40)	2 (22)	12 (26)
Medication dosing	0 (0)	2 (22)	10 (22)
Growth parameters	0 (0)	1 (11)	9 (20)

a Percentages indicate proportion of total errors for each model.

**Figure 1.**
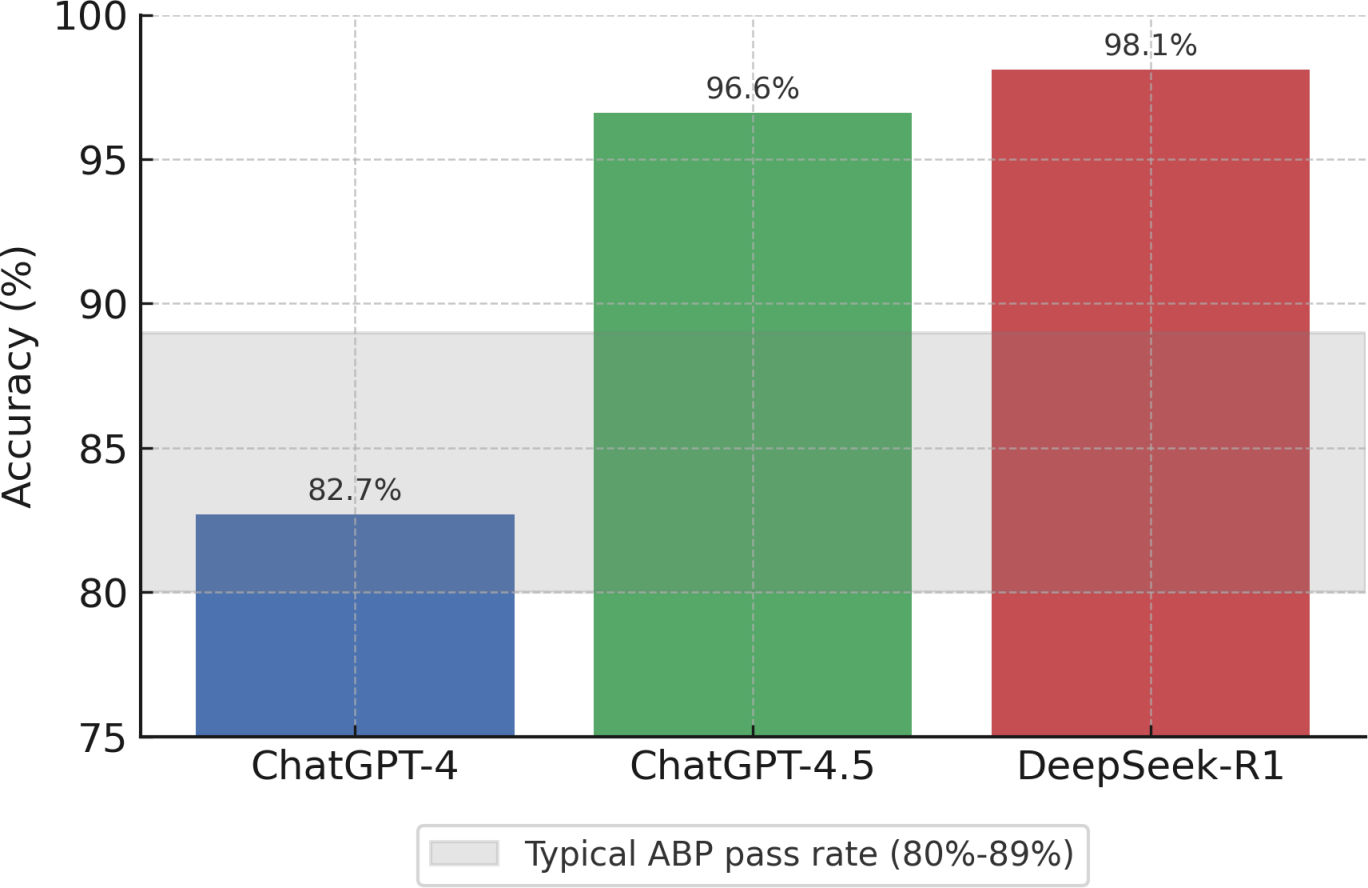
Accuracy of large language models on pediatric board examination preparation questions from the 2023 PREP Self-Assessment. ChatGPT-4, ChatGPT-4.5, and DeepSeek-R1 were each tested on 266 multiple-choice questions. The shaded area represents the typical first-time pass rate range (80%‐89%) for board-certified pediatricians on the ABP examination from 2022 to 2024. DeepSeek-R1 achieved the highest performance at 98.1%, exceeding the typical ABP pass rate range. ABP: American Board of Pediatrics.

These results were compared to the published first-time pass rates for board-certified pediatricians taking the ABP examination, which typically range from 80%‐89% (80% in 2022 and 89% in 2024 for general pediatrics) [5]. As illustrated in Figure 1, DeepSeek’s performance exceeded the typical range for human pediatricians on first-attempt board examinations, while ChatGPT-4.5 also performed at the upper threshold of human performance. ChatGPT-4’s performance was comparable to the lower range of human pass rates.

These findings demonstrate substantial variability in AI model performance on pediatric knowledge assessment, with newer models demonstrating substantial capabilities on pediatric board questions. The following discussion contextualizes these results within the broader landscape of AI in medical education and clinical practice.

## Discussion

Our findings demonstrate that recent advancements in LLMs have produced AI systems capable of performing at or above the level of board-certified pediatricians on standardized examination questions. DeepSeek’s exceptional performance (98.1% accuracy) represents a significant milestone in AI medical knowledge representation, exceeding typical ABP pass rates. The substantial performance gap between AI models highlights the rapid evolution of these technologies, with newer iterations showing marked improvements compared to older versions [4,6].

These results have important implications for medical education, board examination preparation, and potentially clinical decision support. AI models could serve as supplementary educational tools for pediatric trainees, offering accurate content knowledge while human educators focus on clinical reasoning, ethics, and patient communication skills that remain challenging for AI systems [7,8].

AI models could revolutionize medical education through personalized learning pathways, instant feedback on clinical reasoning, and simulation of rare cases [9]. However, critical limitations remain in areas requiring human judgment, empathy, and ethical decision-making. For instance, while AI excels at factual recall, it cannot replicate the nuanced patient interactions, cultural sensitivity, or ethical reasoning essential to pediatric practice [10]. Future applications should focus on AI as a supportive tool that enhances rather than replaces traditional medical education, particularly in areas like case-based learning, differential diagnosis practice, and board examination preparation [11].

Limitations of this study include the use of multiple-choice questions rather than free-response clinical scenarios and the focus on knowledge recall rather than practical clinical decision-making. We cannot determine whether the AI models’ performance reflects true clinical reasoning or pattern recognition based on similar questions in their training data. Additionally, while PREP Self-Assessment questions are designed to simulate board examinations, they may differ in difficulty and content distribution from actual ABP examinations, complicating direct comparisons with human pass rates. Important limitations exist in comparing AI performance to human ABP pass rates. The ABP examination involves 330 questions administered under timed, proctored conditions with associated stress factors, while our AI evaluation used 266 questions without time constraints or test-taking pressure. Additionally, human physicians integrate years of clinical experience, ethical reasoning, and patient interaction skills that are not assessed in multiple-choice formats. Therefore, while our results demonstrate strong knowledge recall by AI models, they should not be interpreted as evidence of superior clinical competence. Furthermore, these models have not been tested on their ability to gather historical information, perform physical examinations, or develop appropriate management plans in real clinical settings [12,13]. Future research should evaluate these AI systems on more complex clinical reasoning tasks and directly compare their performance to practicing pediatricians in simulated clinical scenarios.

## Abbreviations

ABP: American Board of Pediatrics
AI: artificial intelligence
LLM: large language model
